# 1645. Predicting Vancomycin Area Under the Curve by Bayesian-Guided Dosing in Pediatric Patients

**DOI:** 10.1093/ofid/ofad500.1479

**Published:** 2023-11-27

**Authors:** Donghyun Shin, So Eun Park, Soo-Han Choi

**Affiliations:** Pusan National University Hospital, Busan, Pusan-jikhalsi, Republic of Korea; Pusan National University Hospital, Busan, Pusan-jikhalsi, Republic of Korea; Pusan National University School of Medicine, Busan, Pusan-jikhalsi, Republic of Korea

## Abstract

**Background:**

In revised consensus guidelines for therapeutic monitoring of vancomycin, the area under the curve (AUC)-guided monitoring, preferably with Bayesian estimation, is suggested for all pediatric age groups. Recommended AUC target in pediatrics is 400 mg·h/L (potentially up to 600 mg·h/L). However, there is limited pediatric data on validating Bayesian dose-optimizing and predicting vancomycin AUC. We aimed to evaluate the accuracy of the predicted AUC on vancomycin dosing adjustments based on Bayesian estimation.

**Methods:**

We reviewed 25 pediatric patients’ data on vancomycin therapeutic drug monitoring (TDM), including trough concentrations (Ct) and AUC. A Bayesian dose-optimizing software program was used to calculate vancomycin AUC. A second TDM was taken after vancomycin dosing adjustments were performed based on the first TDM. The accuracy was calculated by the ratio of the second AUC to the predicted AUC by Bayesian dose optimization.

**Results:**

The median age of patients was 14.3 years old (interquartile range [IQR], 9.2-16.8). The median vancomycin dosage was 41.7 mg/kg/day (IQR, 33.9-50.0). In the first TDM of vancomycin, the median Ct and AUC were 6.1 ug/mL (IQR, 2.8-11.0; 95% confidence interval [CI], 4.2-9.2) and 320.0 mg·h/L (IQR, 251.5-486.8; 95% CI, 284.0-357.0), respectively. Vancomycin dose increase was required in 68.0% of patients; the median dosage was 50.0 mg/kg/day (IQR, 38.8-60.0). Based on Bayesian-guided dosing adjustments, the median predicted AUC was 420.0 (95% CI, 380.0-447.0). In the second TDM, the median Ct and AUC were 8.7 ug/mL (IQR, 6.2-12.2; 95% CI, 7.5-11.0) and 428.0 mg·h/L (IQR, 369.7-514.0; 95% CI, 384.0-486.0), respectively. The accuracy was 1.033 (95% CI, 0.941-1.307).

**Conclusion:**

The Bayesian AUC-guided dosing strategy may be optimal for individualizing vancomycin therapy in pediatric patients.

**Disclosures:**

**All Authors**: No reported disclosures

